# Acromioclavicular Dislocation Associated with Coracoid Process Fracture: Report of Two Cases and Review of the Literature

**DOI:** 10.1155/2015/858969

**Published:** 2015-09-29

**Authors:** Ozkan Kose, Kerem Canbora, Ferhat Guler, Omer Faruk Kilicaslan, Hasan May

**Affiliations:** ^1^Department of Orthopedics and Traumatology, Antalya Education and Research Hospital, Antalya, Turkey; ^2^Department of Orthopedics and Traumatology, Haydarpasa Numune Education and Research Hospital, Istanbul, Turkey

## Abstract

Acromioclavicular dislocation associated with coracoid process fracture is a rare injury. Herein we reported two further cases with such combination of injuries and reviewed all previously published cases in current literature. In this review, we discussed the demographic characteristics, mechanism of injury, diagnosis, and treatment options extensively.

## 1. Introduction

Isolated acromioclavicular (AC) joint dislocations are frequent injuries that account for approximately 9% of all shoulder girdle injuries [1]. On the other hand, AC joint dislocation associated with coracoid process (CP) fracture is an uncommon combination of injuries. Current literature contains few numbers of published cases, and available knowledge about this rare injury comes from the published case reports. There is no extensive review of these published cases in the relevant literature. Herein, we described two more cases with AC joint dislocation associated with CP fracture and reviewed all previously published cases with such combination of injuries. The purpose of this review is to discuss the demographic and clinical characteristics, mechanism of injury, radiographic features, treatment options, and outcomes regarding AC dislocation associated with CP fracture in light of the current literature.

## 2. Case #1

**Figure 1:**
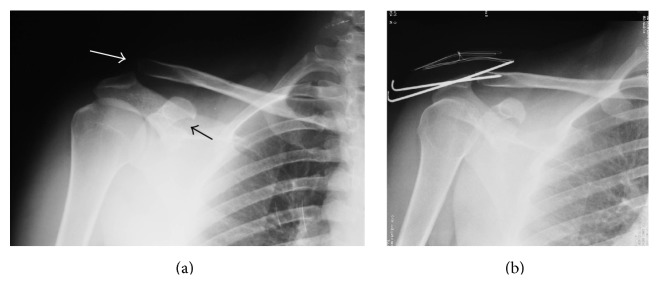


**Figure 2:**
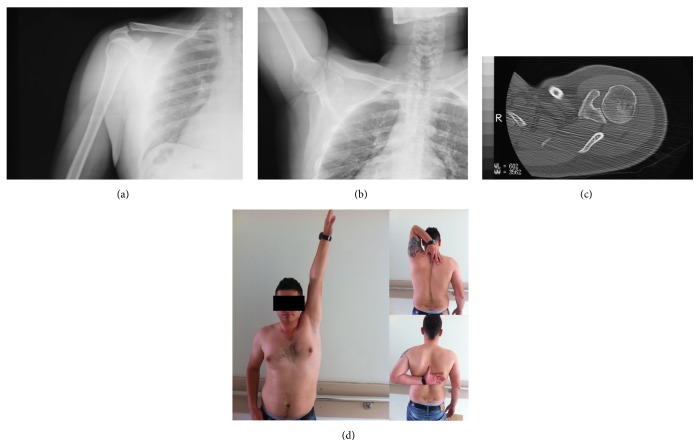


A 24-year-old man (worker) presented to our emergency department with left shoulder pain and deformity after he sustained a motorcycle accident. On physical examination, there was marked prominence over the AC joint which was painful with palpation. Shoulder movements were restricted and painful. Neurovascular examination revealed no abnormalities. Anteroposterior shoulder radiograph demonstrated a Type III AC joint separation with CP fracture (Figure 1(a)). As the patient was a young man who had a hard manual labor, operative treatment was decided. Under general anesthesia in beach chair position, open reduction and percutaneous K wire fixation was performed without additional fixation for CP fracture (Figure 1(b)). Two weeks after the operation, active pendulum exercises were started and gradually increased to full range of shoulder movements. At 5th week, the K wires were extracted. At the final follow-up 8 months after the initial injury, the patient was free of pain with normal shoulder movements and returned back to his previous level of activity. Final radiographs and CT showed union of CP fracture (Figure 2).

## 3. Case #2

**Figure 3:**
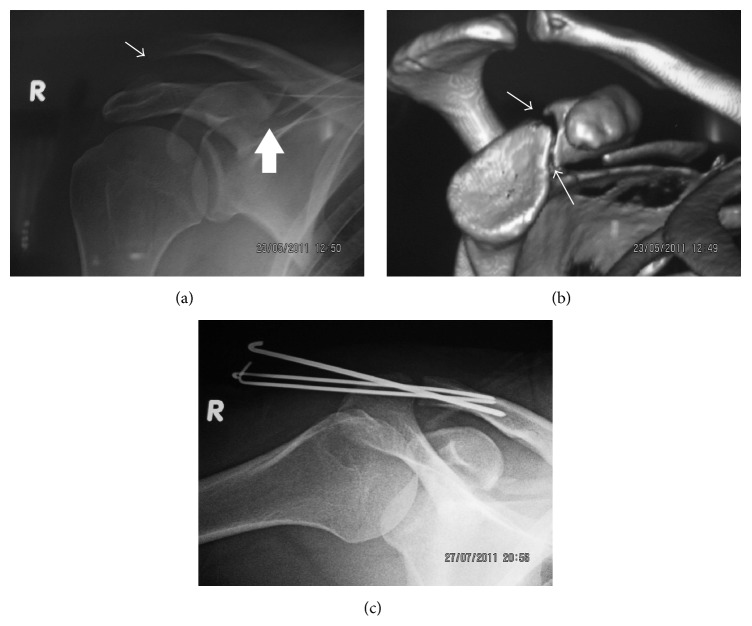


**Figure 4:**
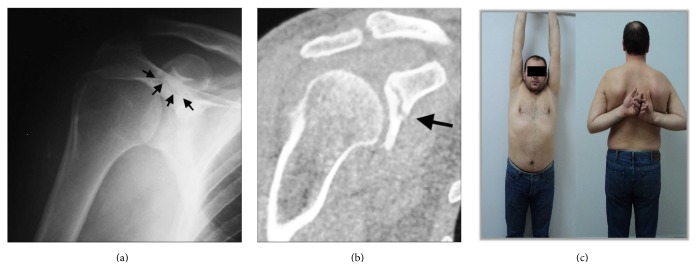


A 36-year-old man (electric technician) was admitted to our emergency department after he fell down from a 2 m height over his right shoulder. On presentation, he complained about isolated shoulder pain. There was a noticeable bump over the AC joint, the so-called the* step-off sign*. Active and passive shoulder movements were painful and the AC joint was tender on palpation. Anteroposterior shoulder X-ray showed Type III AC joint dislocation and there was suspicious findings for a CP fracture (Figure 3(a)). Further CT imaging of the shoulder confirmed the fracture of CP (Figure 3(b)). As the patient had a job involving overhead activity and sustained an injury of his dominant hand, operative treatment was decided. The patient underwent open reduction and percutaneous fixation using multiple K wires transpassing AC joint (Figure 3(c)). No additional fixation was done to CP. The postoperative period was uneventful and the K wires were extracted at the 6th week. At the final follow-up 19 months after the surgery, the shoulder joint movements were full in any direction without pain. He returned to his previous level of activity and work. Final radiographs showed the proper alignment of the AC joint; however CT examination showed the nonunion of the CP fracture (Figure 4). As the patient was asymptomatic and recovered fully, no further surgery was planned.

## 4. Discussion

To the best of our knowledge, a total of 46 previous cases have been reported in the current English literature describing AC joint dislocation associated with CP fracture [2–30] (Table 1). The mean age of the reported cases (missing in three) was 25.4 ± 14.4 years (range, 9–77). Of these cases, 36 (78.3%) were male, 7 (15.2%) were female, and gender was not reported in the remaining 3 (6.5%) patients. The most common etiology was traffic accidents (16 patients, 34.8%) followed by falls (12, 26.1%) and sports trauma (12 patients, 26.1%), respectively. These findings are consistent with the epidemiologic characteristics of the isolated AC joint dislocation in previous studies. This combined injury also is frequent in young male adults.

Two different mechanisms of injury have been proposed for AC dislocations [1, 16, 19, 25]. The first one is direct trauma to the shoulder girdle which is usually caused by a fall or blow with the arm in the adducted position. The second mechanism is indirect trauma which occurs by falling on an adducted outstretched hand or elbow, causing the humerus to translocate superiorly, driving the humeral head into the acromion. In fact the mechanism of injury in combined AC dislocation and CP fractures is the same for isolated AC dislocations, except, instead of disrupting the CC ligaments, a fracture occurs at the coracoid, which allows for vertical displacement of the clavicle [3]. Wilson and Colwill proposed another mechanism of injury for their case and stated that forceful resisted flexion of the arm with resultant strong pull of the conjoint tendon may cause a coracoid fracture [12]. However, this theory can only explain coracoid tip fractures. In contrast, almost all cases with combination injuries had coracoid base fractures.

AC joint is a diarthrodial joint which is stabilized by surrounding strong ligaments including acromioclavicular ligaments (superior, inferior, anterior, and posterior), coracoclavicular ligaments (trapezoid and conoid), and coracoacromial ligament [1]. CP is a small hook-like structure that acts as a strong attachment point for the short head of biceps, coracobrachialis, and pectoralis minor muscles [19, 21, 24, 25]. Thus relatively high energy trauma is required for combination of injuries to occur. Similarly, we have identified that almost one out of three patients (34.1%) sustained traffic accidents.

Diagnosis of an AC dislocation is usually made with direct shoulder radiographs as the clinical findings are often evident particularly in complete AC dislocations (Type III and over), since the distance between the clavicle and CP widens and the AC joint congruity is disrupted [1, 22]. On the other hand CP fracture may be missed due to its complex anatomic structure and superimposition on standard shoulder radiograph [9, 25]. In case of a simultaneous injury to AC joint and CP, it may be easy to miss a CP fracture that is overshadowed by more obvious AC dislocation [25]. A high degree of suspicion is necessary and physicians should keep in mind that these fractures can occur simultaneously. Besides standard shoulder radiographs (anteroposterior, lateral, and axillary view), special radiographic views have been described to visualize the CP better, such as Zanca view and Stryker notch view [1, 19, 21, 25, 27]. Zanca view is a modified anteroposterior view in which the X-ray beam is directed at the AC joint with 10-degree cephalic tilt. This eliminates the overlapping bony structure and demonstrates the AC joint better. Stryker notch view is taken when the affected extremity is placed on the top of the head and the X-ray beam is directed from anterior to posterior towards coracoid with 10-degree cephalic tilt. This projection provides a better view of the coracoid process. However, in emergency settings, patient positioning may be difficult due to pain. In case of suspicious radiographic findings, further CT examination clearly depicts the fracture [18, 20]. CT, particularly 3D reconstruction, is beneficial to realize the spatial configuration of the fracture and its extensions.

**Figure 5:**
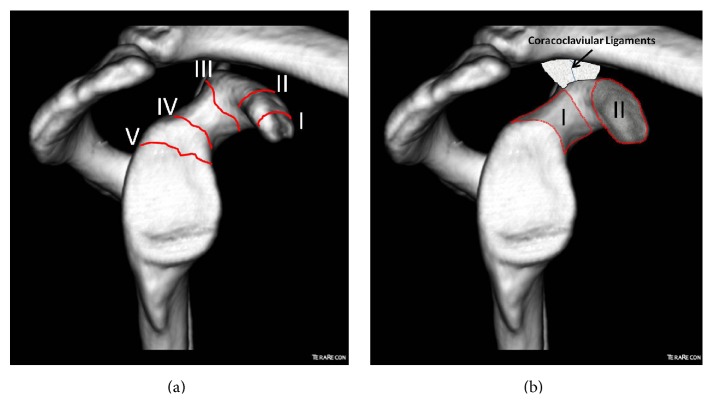


Currently there are two different classification systems for isolated CP fractures. In 1995, Eyres et al. first classified these fractures into 5 types according to the fracture location, namely, tip avulsions (1), mid-process fractures (2), basal fractures (3), superior scapula fractures (4), and fractures extending to glenoid articular surface (5) [18] (Figure 5(a)). Later on, Ogawa et al. proposed a new classification system. They divided the CP into two distinct locations based on the CC ligament attachment; namely, Type I fractures were located behind these ligaments and Type II ones were in front of them [31] (Figure 5(b)). In a combined injury AC joint and coracoids process, the fracture pattern of the coracoid is usually Type I fracture according to Ogawa and Types III and IV fracture according to Eyres et al. [18]. Traction forces mediated by CC ligaments may be the reason for this specific fracture pattern [8, 18].

A wide variety of treatment options have been reported for this combination of injuries ranging from conservative treatment in the form of shoulder sling [5] to complex surgical reconstructions and fixation. Treatment options can be divided into two groups either conservative treatment or surgical intervention. There is no consensus regarding the preferred treatment for these injuries and factors that contribute to the decision making process include associated injuries, patient activity level and occupation, and hand dominance. According to authors who advocate surgical treatment, this combined injury causes a double disruption of the superior shoulder suspensory complex; thus an unstable fracture which may compromise the integrity of the linkage between the clavicle and the scapula occurs [6, 16, 19, 22]. Secondly, CP is a strong attachment point for several muscles. Therefore, these muscular forces may cause further displacement and motion resulting in nonunion. As a third argument, early range of motion and returning back to normal physical activities is possible with rigid fixation.

Almost half of the reported cases have been treated conservatively and result in good and excellent outcome [2, 3, 7, 9, 10, 13–15, 17, 21, 26, 29]. According to our literature review, it is hard to suggest best treatment option. Similarly, treatment of isolated Type III AC dislocations is still contradictory. In a recent review, there was no clear advantage of surgery over conservative treatment, and functional outcomes were similar [32]. Some authors [3, 4, 8, 9] advocated surgical fixation of only one disruption point (usually the AC joint), and some authors [11, 12, 16, 19, 20, 23–25, 28] performed surgical intervention on both disruption points (i.e., fixation of CP fracture and AC dislocation). In our cases, we preferred surgical fixation of the AC joint only. One of our patients had pseudoarthrosis of the CP fracture at the final follow-up, although his clinical outcome was excellent.

In conclusion, AC dislocation associated with CP fracture is a rare injury. This combined injury is usually seen in young male adults who sustained high energy trauma. Diagnosis may be difficult on standard shoulder radiographs, and special views and/or CT can be used for definitive diagnosis in case suspicion. The treatment is still controversial; both conservative and surgical treatment methods seem to be equally effective.

## Figures and Tables

**Table 1:** Previously published cases of AC dislocation associated with CP fracture in English literature (M: male, F: female, and K: Kirschner).

Author	Case #	Year	Age	Sex	Mechanism of injury	Treatment		Functional outcome
						Coracoid fracture	AC dislocation	
Urist [2]	1	1946	11	M	Traffic accident	Conservative		Good
	2		?	M	Fall	Conservative		Good
Protass et al. [3]	3	1975	17	M	Sport trauma	Conservative		?
	4		22	F	Traffic accident	None	AC joint K wiring	?
	5		14	M	Fall	Conservative		?
Smith [4]	6	1975	?	M	Fall	None	AC joint K wiring	Good
Zettas and Muchnic [5]	7	1976	13	M	Sport trauma	Screw fixation	CC ligament repair	Excellent
	8		26	F	Fall	Screw fixation	None	Excellent
Montgomery and Loyd [6]	9	1977	15	M	Sport trauma	Suture fixation	None	Excellent
	10		15	M	Traffic accident	Conservative		Poor
Lasda and Murray [7]	11	1978	51	M	Traffic accident	Conservative		Good
Ishizuki et al. [8]	12	1981	18	M	?	None	AC joint K wiring	Good
	13		29	M	Traffic accident	Dewar-Barrington procedure		Good
	14		48	F	Traffic accident	Dewar-Barrington procedure		Good
	15		27	M	Fall	Dewar-Barrington procedure		Good
Bernard et al. [9]	16	1983	13	M	Sport trauma	Conservative		Good
	17		15	M	Sport trauma	Conservative		Excellent
	18		17	M	Traffic accident	None	AC joint K wiring	Good
	19		28	M	Traffic accident	Conservative		Good
Taga et al. [10]	20	1986	9	F	Fall	Conservative		Good
Barentsz and Driessen [11]	21	1989	19	M	Sport trauma	Screw fixation	AC joint tension band wiring	Good
Wilson and Colwill [12]	22	1989	20	M	Sport trauma	Screw fixation	Screw fixation and Dacron loop	Excellent
Carr and Broughton [13]	23	1989	46	M	Traffic accident	Conservative		Excellent
	24		23	F	Fall	Conservative		Excellent
Martín-Herrero et al. [14]	25	1990	16	M	Sport trauma	Conservative		Excellent
	26		24	M	Traffic accident	Conservative		Excellent
	27		17	M	Sport trauma	Conservative		Excellent
	28		60	M	Traffic accident	Conservative		Good
Hak and Johnson [15]	29	1993	24	M	Bicycle accident	Conservative		Excellent
Wang et al. [16]	30	1994	28	M	Traffic accident	Screw fixation	AC joint K wiring, Marlex loop	Excellent
Combalia et al. [17]	31	1995	12	M	Sport trauma	Conservative		Excellent
Eyres et al. [18]	32	1995	27	F	Assault	Screw fixation		Excellent
	33		?	?	?	?	?	?
	34		?	?	?	?	?	?
Yu et al. [19]	35	2002	37	M	Traffic accident	Screw fixation	AC joint K wiring, CC ligament repair	Excellent
Güneş et al. [20]	36	2006	30	M	Fall	Screw fixation	Screw fixation	Excellent
DiPaola and Marchetto [21]	37	2009	15	M	Sport trauma	Conservative		Excellent
Kim et al. [22]	38	2009	?	?	?	Screw fixation		?
Duan et al. [23]	39	2010	40	M	Traffic accident	Screw fixation	Clavicular hook-plate fixation	Excellent
Jettoo et al. [24]	40	2010	12	M	Fall	Screw fixation	AC joint K wiring	Excellent
Li et al. [25]	41	2010	37	M	Fall	Screw fixation	Clavicular hook plate	Excellent
Thomas et al. [26]	42	2010	22	F	Fall	Conservative		Excellent
Bhatia [27]	43	2012	77	M	Fall	Screw fixation		Excellent
Kawasaki et al. [28]	44	2014	33	M	Traffic accident	Screw fixation	Clavicular hook plate	Excellent
Pedersen et al. [29]	45	2014	14	M	Sport trauma	Conservative		Excellent
Naik et al. [30]	46	2015	24	M	Traffic accident	Screw fixation	AC joint K wiring	Excellent
Current cases	47	2015	21	M	Traffic accident	None	AC joint K wiring	Excellent
	48	2015	34	M	Traffic accident	None	AC joint K wiring	Excellent
